# Vitamin D Deficiency in Pregnant Women and Their Infants

**DOI:** 10.4274/jcrpe.4706

**Published:** 2018-02-26

**Authors:** Abdurrahman Avar Özdemir, Yasemin Ercan Gündemir, Mustafa Küçük, Deniz Yıldıran Sarıcı, Yusuf Elgörmüş, Yakup Çağ, Günal Bilek

**Affiliations:** 1 Biruni University, Medicine Hospital, Clinic of Pediatrics, İstanbul, Turkey; 2 Biruni University, Medicine Hospital, Clinic of Obstetrics and Gynecology, İstanbul, Turkey; 3 Kartal Dr. Lütfi Kırdar Training and Research Hospital, Clinic of Pediatrics, İstanbul, Turkey; 4 Bitlis Eren University Faculty of Arts and Sciences, Department of Statistics, Bitlis, Turkey

**Keywords:** Vitamin D, neonate, pregnancy

## Abstract

**Objective:**

Vitamin D deficiency is a serious health problem despite a general improvement in socio-economic status in Turkey. The aim of this study was to evaluate maternal vitamin D status and its effect on neonatal vitamin D concentrations after a support programme for pregnant women was introduced. A second aim was to identify risk factors for vitamin D deficiency in a district of İstanbul.

**Methods:**

A total of 97 pregnant women and 90 infants were included in this study, conducted between January and October 2016. The demographic data, risk factors and daily vitamin intake were recorded. Serum levels of vitamin D, calcium, phosphorus and alkaline phosphatase in all subjects were measured. The mothers and newborns were divided into groups based on their vitamin D levels. The relationship between vitamin D levels and risk factors was analyzed.

**Results:**

Mean ± standard deviation vitamin D levels for the women and their infants were found to be 14.82±11.45 and 13.16±7.16 ng/mL, respectively. The number of mothers and infants was significantly higher in the deficient group, and their mean vitamin D levels significantly lower (9.02±1.34 and 8.80±1.06 ng/mL, respectively) (p<0.001, p<0.001). Only 14.4% of pregnant women took 1000-1200 IU/day of vitamin D. When the mother groups were evaluated in terms of risk factors, there were significant differences in daily vitamin intake and clothing style (p<0.001 and p<0.001 respectively).

**Conclusion:**

Vitamin D deficiency in pregnant women and their infants is still a serious health problem in Turkey, although a vitamin D support programme during pregnancy has been launched by the department of health.

## What is already known on this topic?

Despite improvement in the socio-economic level of the population, vitamin D deficiency is still a serious health problem in Turkey. A new vitamin D support programme was launched for pregnant women by the Ministry of Health in 2011.

## 

### What this study adds?

Despite the launch of a vitamin D support programme for pregnant women by the Ministry of Health, vitamin D deficiency in pregnant women and their infants continues to be a serious health problem in Turkey.

## Introduction

Vitamin D is not only a lipid-soluble vitamin, but also a steroid hormone that can be synthesized endogenously. It has an important role in calcium (Ca)-phosphorus (P) homeostasis and its deficiency causes rickets in children and osteomalacia in adults (1,2). Vitamin D deficiency may also result in impairment of immune function, predisposition to cancer, cardiovascular disease, diabetes, rheumatic disease, muscle weakness, chronic pain and neuropsychiatric dysfunction (3,4,5,6,7). The lack of vitamin D during pregnancy is the most important risk factor for infantile rickets and may also result in poor fetal growth and neonatal development (8,9,10,11). In addition, its deficiency in pregnant women may predispose to gestational diabetes mellitus and preeclampsia (12,13). 

Vitamin D deficiency continues to be a serious health problem in Turkey despite a general improvement in socio-economic status in recent years. In 2005, a “Vitamin D prophylaxis augmentation program” was initiated by the Turkish Pediatric Endocrine Society and the Ministry of Health for prevention of rickets. This program included free distribution of supplements to provide 400 IU/day of vitamin D. At the end of this program, the prevalence of rickets decreased in children under three years of age (14). After this success, a new nationwide vitamin D support program was launched for pregnant women by the Ministry of Health in 2011. This program included 1200 IU vitamin D replacement daily to all pregnant women from the first trimester of pregnancy until six months after delivery (15). 

The aim of this study was to evaluate maternal vitamin D status and its effect on neonatal vitamin D status, following the introduction of the support programme for pregnant women. A second aim was to identify risk factors for vitamin D deficiency in the Istanbul district of Bağcılar, a low socio-economic neighborhood.

## Methods

This prospective study was conducted in cooperation with the Departments of Pediatrics and Obstetrics and Gynecology in Medicine Hospital/Biruni University. The study protocol was approved by the Ethics Committee of the Biruni University (approval number: 2015-KAEK-43). Informed consent was obtained from pregnant women and given by them for the participation of their infants.

It was planned to include one hundred and twenty women in their 3^rd^ trimester of pregnancy and their infants in this study, but 23 women were excluded as they rejected inclusion of their babies. Also, seven infants were excluded from this study because blood samples could not be obtained. Women younger than 20 or over 40 years of age, those with chronic disease, taking medications and those with twin pregnancy were excluded. Being small for gestational age (SGA; defined as a birth weight less than 2500 g), prematurity, having a congenital disease or malformation, age at sampling older than 28 days and refusal of parental consent were exclusion criteria for infants. Thus, a total of 97 pregnant women and 90 infants were included in this study conducted between January and October 2016. Information on the mothers and their infants such as age, gender, weight, height, parity, socio-economic status, daily sun exposure, daily vitamin D intake, style of clothing and season were recorded. Not being exposed to sunlight daily was defined as low sun exposure for the mothers. Using a headscarf and clothes which covered arms and legs were defined as covered clothing style. The mothers were divided into three groups according to daily vitamin D intake; none, 400-600 IU/day and 1000-1200 vitamin D IU/day.

Body mass index (BMI) was calculated by the formula [weight (kg)/height (m)^2^]. Blood samples for (Ca), P, alkaline phosphatase (ALP) and 25-hydroxyvitamin (OH) D were taken from the mothers within one month, prior to delivery and from the infants within one week after delivery. Samples were examined on the same day. The 25(OH)D levels were measured by enzyme linked fluorescent assay on the Mini Vidas (Biomerieux, France). Ca, P and ALP were measured using photometry on the Cobas Integra 400 Plus (Roche Diagnostics, Germany). Participants were divided into three groups as deficient, insufficient or sufficient according to their vitamin D levels. 25(OH)D levels were defined as <12 ng/mL (<30 nmol/L) deficient, 12-20 ng/mL (30-50 nmol/L) as insufficient and >20 ng/mL (>50 nmol/L) as sufficient (16).

In this study, IBM SPSS v20 and R were used to conduct the analysis. The Statistical G Power program was used to calculate sample size. We estimated a minimum total sample size of 84 to achieve an effect size of 0.35, the power of 0.8 and type 1 error of 0.05. Descriptive statistics are given via tables. Chi-square test of independence was used to detect the significant relationships between nominal variables. To test the differences between means, t-test, one-way ANOVA for normally distributed data and Mann-Whitney U test and Kruskal-Wallis H test for nonparametric data were used. To detect from which groups the difference originated, Tukey’s honestly significant difference and Dunn’s tests were used.

## Results

Ninety-seven pregnant women were included in this prospective study. The mean vitamin D level for all women was 14.82±11.45 ng/mL. When the risk factors were evaluated in pregnant women, there were statistically significant differences in BMI, daily vitamin intake and clothing style (p=0.02, p<0.001, p=0.02 respectively). The characteristics of the groups are shown in Table 1.

The number of women was significantly higher in the deficient group (p<0.001), and their mean vitamin D level was significantly lower (9.02±1.34 ng/mL) than insufficient (15.13±2.34 ng/mL) and sufficient groups (33.95±20.71 ng/mL) (p<0.001; see Table 2). No significant differences were found between groups in terms of Ca, P or ALP levels (p=0.07 p=0.10**,** p=0.94). When the groups were evaluated in terms of risk factors, there were statistically significant differences in daily vitamin intake and clothing style (p<0.001, p<0.001).

Ninety infants were included in this prospective study. Mean vitamin D level was 13.16±7.16 ng/mL for the total group of infants. The mean gestational age and birth weight of infants were 38.45±1.10 weeks and 3.36±0.39 kg respectively. The number of female infants was 48 (53%). Infants were divided into three groups according to their vitamin D levels. The number of infants in the deficient group was significantly higher than that in insufficient and sufficient groups (p<0.001). Mean vitamin D level in the deficient group was 8.80±1.06 ng/mL and this level was significantly lower when compared to the insufficient (15.43±2.33 ng/mL) and sufficient groups (28.84±9.26 ng/mL; p<0.001). Among the groups, there were no differences in Ca, P and ALP levels (Table 3).

When the effect of maternal risk factors on their infants’ vitamin D levels was evaluated, there were no statistical differences, with the exception of daily vitamin D intake. Mean vitamin D levels of babies whose mothers wear covered clothing or not were 13.01 and 13.44 ng/mL, respectively. This difference is statistically insignificant (Independent sample t-test, p=0.79). Mean vitamin D level in infants whose mothers took no daily vitamin D, in infants whose mothers took 400-<1000 IU and 1000-1200 IU daily were 12.13, 12.95 and 16.25 ng/mL, respectively. There were statistically significant differences in the mean values of these three groups (Kruskal-Wallis H test, p=0.04), and this difference originated from the 400-<1000 IU and 1000-1200 IU groups (Dunn’s test, p=0.01).

Also, we evaluated the infants according to mother’s vitamin D status. Mean vitamin D level in the infants of mothers who have deficient, insufficient and sufficient vitamin D levels were 10.05±3.70 ng/mL, 13.06±4.09 ng/mL and 24.28±10.33 ng/mL, respectively (p<0.001) (Table 4). The Pearson correlation between the mothers’ and their babies’ vitamin D levels was significant (p<0.001) and the correlation coefficient was 0.63.

## Discussion

Vitamin D deficiency leads to important health problems, not only in mothers but also in their infants, because the vitamin D store of the mother is the major source of vitamin D for the fetus (9). The vitamin D dose that the World Health Organization recommends for pregnant women is 200 IU/day (17). The Institute of Medicine suggested that the “Estimated Average Requirement” and “Recommended Dietary Allowance” (RDA) for pregnant women be 400 and 600 IU/day, respectively (18). Recent studies reported that the daily dose for pregnant women should be greater than 1000 IU/day to achieve adequate levels (8,19). The safety dose during pregnancy is not clear, but Hollis et al (20) showed that vitamin D supplementation of 4000 IU/day for achieving adequate levels was safe and effective in pregnant women.

The International Association of Endocrinology defined a vitamin D level of 21-29 ng /mL as insufficiency and <20 ng/mL as deficiency in adults (21). However, the levels of vitamin D insufficiency and deficiency are not clearly defined and the discussion about the prevalence vitamin D deficiency is ongoing (22,23). The recommended value for serum vitamin D level is lower in children than adults. The Endocrine Society suggests vitamin D levels of > 20 ng/mL for sufficiency, 12-20 ng/mL for insufficiency and <12 ng/mL as vitamin D deficiency (16). This recommendation was used in our study.

Studies reported from different countries have shown a prevalence of vitamin D deficiency in pregnant women and in infants ranging from 4% to 60% and from 3% to 86%, respectively (24,25). In a study from Egypt, El Koumi et al (26) reported that only 35.8% of pregnant women had blood levels over 20 ng/mL. In a study from India, it has been reported that 84% of pregnant women had vitamin D concentrations <22.5 ng/mL (27). In a national survey from Belgium, vitamin D insufficiency (<30 ng/mL) and deficiency (<20 ng/mL) were found as 74.1% and 44.6% (28).

Previous studies have shown that vitamin D deficiency is common in pregnant women in Turkey. In 1998, Alagöl et al (29) found that vitamin D levels were low in 66.6% of women of reproductive age in İstanbul. In 2003, Pehlivan et al (30) reported that 94.8% of the mothers and 24.6% of their infants had levels below 16 ng/mL. In a further study by Ergur et al (2009) (31), only 18.6% of the mothers and 2.9% of the neonates had normal vitamin D levels. In 2008, Halicioglu et al (32) found that 50.4% of pregnant women in İzmir, a city in a sunny region of Turkey, had blood vitamin D levels ≤10 ng/mL. In a study conducted in Ankara in 2010, vitamin D deficiency (≤20 ng/mL) in pregnant women and their infants were found to be 62.6% and 58.6%, respectively (33). It should be noted that all of these studies were conducted prior to the introduction of the national pregnancy vitamin D supplementation programme. In this present study, mean vitamin D level was found to be 14.82±11.45 ng/mL in pregnant women and 13.16±7.16 ng/mL in their infants. Vitamin D deficiency in mothers and infants were 49.5% and 56.7%, respectively. All these data confirm that vitamin D deficiency continues to be a problem in pregnant women and their infants in Turkey, despite the introduction of the supplementation programme. 

Although the Ministry of Health has recommended a vitamin D intake of 1200 IU/day, we found that 12.4% of mothers never used vitamin D supplements and 73.2% used irregular or low doses. The proportion of pregnant women who received 1000-1200 IU/day of vitamin D was 14.4% and this low value was statistically significant. Vitamin D levels were significantly lower in mothers who used low dose vitamin D supplements compared to those who used recommended doses. These results show that high dose vitamin D support is necessary during pregnancy and that the support program should be continued. 

Limited sun exposure, regular use of sunscreens, living in northern latitudes, dark skin, obesity, extensive clothing cover, aging, poor nutritional status, malabsorption syndromes and medications have been reported as risk factors for vitamin D deficiency (1,19). In previous studies in Turkey, winter season, low socioeconomic status, low educational status and covered clothing style were reported as risk factors for vitamin D deficiency in mothers (31,34,35). However, Pehlivan et al (30) found no significant difference related to factors other than covered clothing. Similarly, Halicioglu et al (32) and Çuhacı-Çakır et al (36) reported that covered clothing style was a risk factor for vitamin D deficiency. In this present study, the difference was not in terms of socioeconomic status and season because all mothers had low or moderate incomes and only five (5.2%) of mothers gave birth in winter. We found no differences in terms of number of parity and sunlight exposure, but vitamin D levels of mothers who had covered clothing had significantly lower blood levels than the uncovered women. These findings show that covered clothing style is an important factor for vitamin D deficiency in pregnant women in Turkey.

When we evaluated the infants according to the vitamin D levels of their mothers, we found no difference between groups in terms of gender, gestational age, birth weight, delivery route and the levels of Ca, P and ALP. However, the infants of mothers in the sufficient group had significantly higher vitamin D level than other infants. As might be expected previous studies have shown a strong correlation between maternal and neonatal levels of vitamin D (8,9). Ergur et al (31) suggested that maternal deficiency was the most important factor for vitamin D deficiency in newborns. Andiran et al (35) reported that the most important risk factor for low level in the newborn was a maternal 25(OH)D level below 10 ng/mL. Similarly, we found a strong positive correlation between the mothers’ and their babies’ vitamin D levels and low level of vitamin D in mother was an important risk factor for deficiency in infants. 

When we evaluated the relationship between the infants’ vitamin D level and their mothers’ clothing style, together with daily vitamin D intake, we found that there were no significant differences with respect to the mothers’ clothing style. However, mothers’ low vitamin intake was found as a risk factor for the infants’ vitamin D level.

### Study Limitations

Our study has some limitations that should be mentioned. First, this study was conducted in a single district of İstanbul and second, this district has a population of low socio-economic level. Therefore, this study may be insufficient to evaluate all socio-economic levels and all other regions in Turkey. Further studies are needed to evaluate the limitations of this study.

## Conclusion

Although a vitamin D support programme was launched for pregnant women by the Ministry of Health in 2011, vitamin D deficiency in pregnant women and their infants is still a serious health problem in Turkey in 2016. Also, the data from this study indicate that the usage rate of the dose recommended by the support programme was very low and the prescribed supplements were generally multivitamin preparations. Therefore, the support programme should be continued, more widely promoted and physicians should be more informed about the content of the support programme in pregnancy.

## Figures and Tables

**Table 1:**
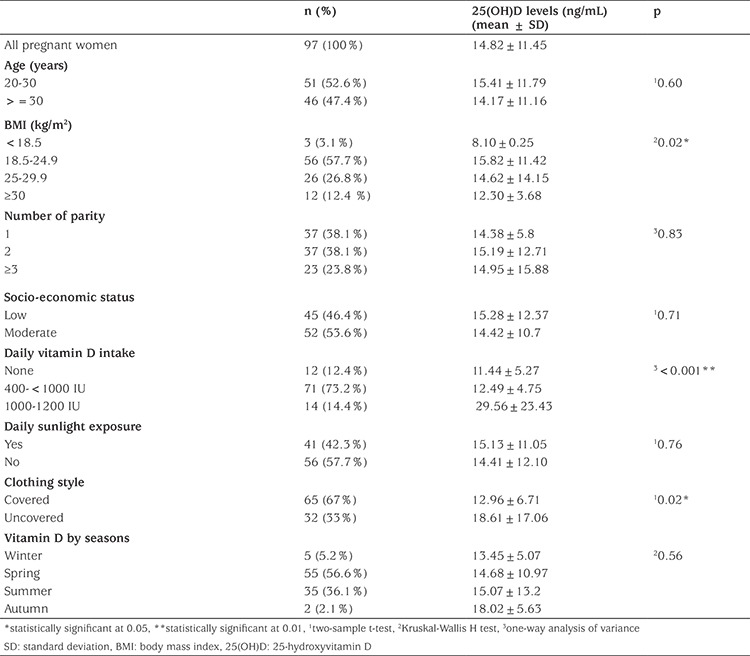
Serum 25-hydroxyvitamin D levels in pregnant women according to their characteristics

**Table 2:**
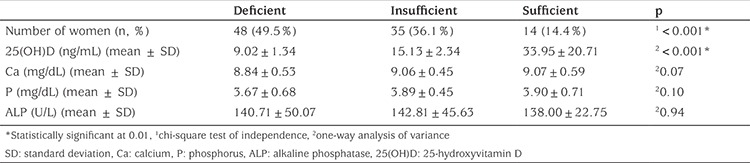
Maternal groups and their laboratory results by vitamin D status

**Table 3:**
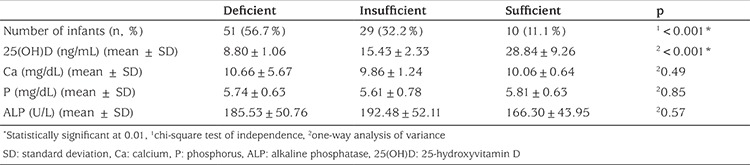
Infant groups and their laboratory results by vitamin D status

**Table 4:**
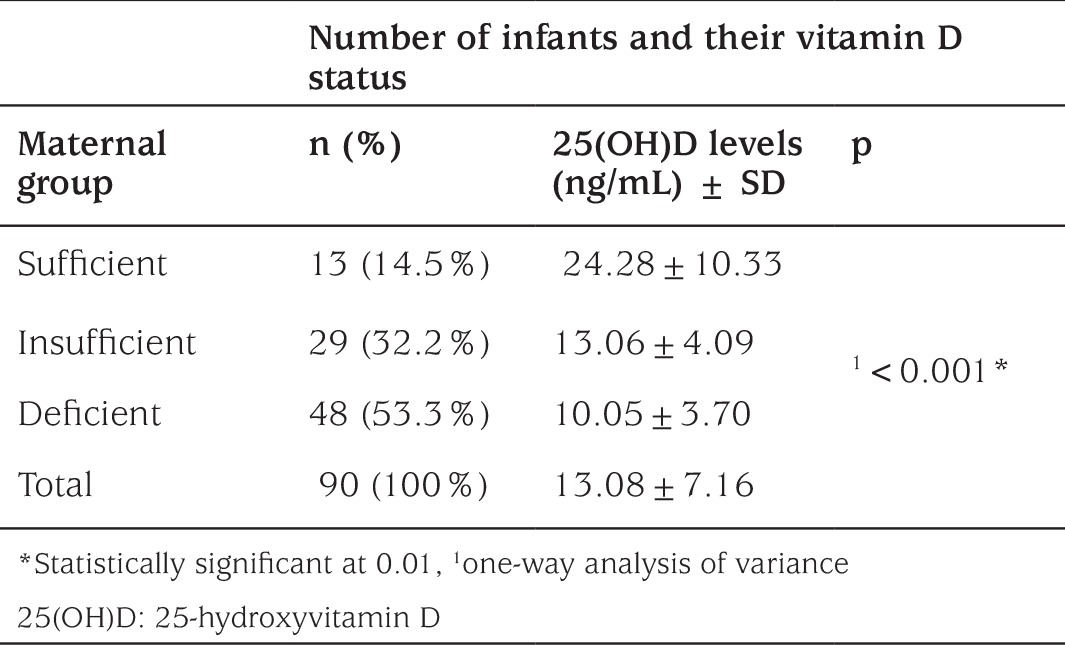
Number of infants and their vitamin D status according to maternal groups
